# Pain as an underrecognized geriatric syndrome in hospitalized older adults with major neurocognitive disorder: clinical correlates and outcomes

**DOI:** 10.1007/s41999-026-01450-w

**Published:** 2026-03-10

**Authors:** Francesca Mancinetti, Emma Giulia Travaglini, Ludovica Speziali, Martina Gaspari, Sara Ercolani, Patrizia Mecocci, Virginia Boccardi

**Affiliations:** 1 Division of Gerontology and Geriatrics, Department of Medicine and Surgery, University of Perugia, Piazzale Gambuli 1, 06132 Perugia, Italy; 2 Division of Clinical Geriatrics, Department of Neurobiology, Care Sciences and Society, Karolinska Institutet, Stockholm, Sweden

**Keywords:** Dementia, Frailty, Geriatrics, Hospital, Mortality, Older, Pain

## Abstract

**Aim:**

This study aims at investigating pain as an underrecognized geriatric syndrome in hospitalized older adults with major neurocognitive disorder, and to evaluate its association with frailty, quality of life, and short-term mortality.

**Methods:**

This prospective observational cohort study was conducted in an acute geriatric care unit. Dementia was diagnosed according to DSM-5 criteria and staged using the Clinical Dementia Rating (CDR). Pain was assessed with the Pain Assessment in Advanced Dementia (PAIN-AD) scale. Frailty (Clinical Frailty Scale) and quality of life (EQ-5D-3L) were evaluated. Analyses included correlation, linear and Cox regression, and Kaplan–Meier survival methods.

**Results:**

A total of 292 hospitalized older adults with major neurocognitive disorder were included (mean age 87.8 ± 6.2 years; 63.4% women). Clinically relevant pain-related behaviours (PAIN-AD ≥ 2) were observed in 62.4% of participants and increased progressively with dementia severity. Despite this high prevalence, only 20.3% of patients with PAIN-AD ≥ 2 were receiving pharmacological analgesic treatment at hospital admission. Compared with participants with PAIN-AD ≤ 1, those with PAIN-AD ≥ 2 showed lower functional autonomy, poorer nutritional status, higher frailty levels, greater acute clinical severity, and longer hospital stay. PAIN-AD scores were positively associated with frailty and poorer health-related quality of life, controlled for multiple covariates. At 3-month follow-up, mortality was significantly higher among patients with PAIN-AD ≥ 2 (40.1% vs 16.3%). In Cox regression analysis, PAIN-AD ≥ 2 remained independently associated with increased short-term mortality (HR 2.39, 95% CI 1.20–4.74), after adjustment for demographic factors, dementia severity, nutritional status, acute illness severity, and analgesic therapy.

**Conclusions:**

Pain assessed by observational tools is frequent and clinically relevant in hospitalized older adults with major neurocognitive disorder and is associated with increased vulnerability and adverse short-term outcomes.

## Key summary points

**Aim:**

To examine pain as a geriatric syndrome in hospitalized older adults with cognitive impairment and assess its prevalence, clinical correlates, and impact on frailty, quality of life, and short-term mortality.

**Findings:**

Higher PAIN-AD scores correlated with greater frailty, poorer quality of life, and higher short-term mortality. PAIN-AD remained independently associated with frailty, reduced quality of life, and mortality in adjusted models. Survival was significantly lower in individuals with higher PAIN-AD scores.

**Message:**

Pain in dementia is frequently overlooked and minimally treated, yet strongly linked to vulnerability and adverse outcomes. Routine use of PAIN-AD may improve detection and management in geriatric care.

## Introduction

Pain is increasingly recognized as a critical health concern among older adults, particularly those with major neurocognitive disorder [1]. Despite its high prevalence, it remains underdiagnosed and undertreated in this population due to cognitive, sensory, and communicative impairments that hinder self-reporting [2]. In the context of advanced cognitive impairment, pain often manifests through behavioural and neurovegetative signs rather than verbal expression, leading to frequent under-recognition in acute care settings [3].

The overlap between neurodegeneration, pain perception, and behavioral symptoms suggests that pain in individuals with dementia should be conceptualized not merely as a comorbidity, but as a multidimensional clinical condition requiring specialized assessment and management strategies [4]. The concept of geriatric syndromes includes clinical conditions that arise from the interaction of age-related vulnerabilities, multimorbidity, and polypharmacy, leading to increased functional decline, and adverse health outcomes, including reduced quality of life and mortality [5]. Pain fulfills several defining features of geriatric syndromes, as it is highly prevalent, multifactorial, frequently under-recognized, and strongly associated with functional impairment, frailty, and adverse outcomes [6]. Indeed, pain has been associated with reduced mobility, cognitive deterioration, increased behavioural disturbances [7], and a heightened risk of mortality [8] in older adults [7, 9]. Pain assessment remains challenging, as traditional self-report tools are often inapplicable in individuals with moderate-to-severe cognitive impairment [3]. For this reason, observational pain assessment instruments have been developed to capture pain-related behaviours [3]. Among them the Pain Assessment in Advanced Dementia (PAIN-AD) scale [10], offers a promising approach to evaluating pain in non-verbal patients, yet their clinical utility and prognostic significance remain underexplored. The PAIN-AD is designed to assess the presence and intensity of observable pain-related behaviours at the time of evaluation, and has been validated in several care settings, including long-term care facilities [11] and community environments [12], while few are the evidence in acute geriatric setting [13]. A recent multi-centre observational study including 181 older people (≥ 65 years) showed that the PAINAD has potential as an effective pain assessment tool in emergency departments [14]. Another more recent study from a longitudinal study of 230 people with dementia admitted to two acute general hospitals showed inter‐rater reliability and internal consistency. Overall this study raises concerns about the validity of the PAINAD in general acute hospitals [15].

In light of this limited and sometimes contradictory evidence, the aim of our study is to better characterize pain as a geriatric syndrome in older persons with cognitive impairment by examining its prevalence, clinical correlates, and short term impact on negative outcomes in a population of hospitalized persons. Understanding the role of pain detected through observational tools in this vulnerable population is essential to improve clinical recognition, guide appropriate management strategies, and ultimately improve patient-centred outcomes.

## Methods

### Participants and study design

This study is a single-centre prospective observational cohort study conducted in older adults (≥ 65 years) with major neurocognitive disorder admitted to the acute Geriatric Care Unit of Santa Maria della Misericordia Hospital (Perugia, Italy) in the period between May 2024 and December 2024. The inclusion criteria included diagnostic evidence of major neurocognitive disorder (according to DSM-V criteria [16]). Cognitive impairment severity was classified using the Clinical Dementia Rating (CDR) scale, allowing the inclusion of patients with mild, moderate, and severe dementia. It assesses cognitive and functional decline in multiple domains: memory, orientation, judgment and problem-solving skills, community relations, home and hobbies, and personal care [17, 18].

**Fig. 1:**
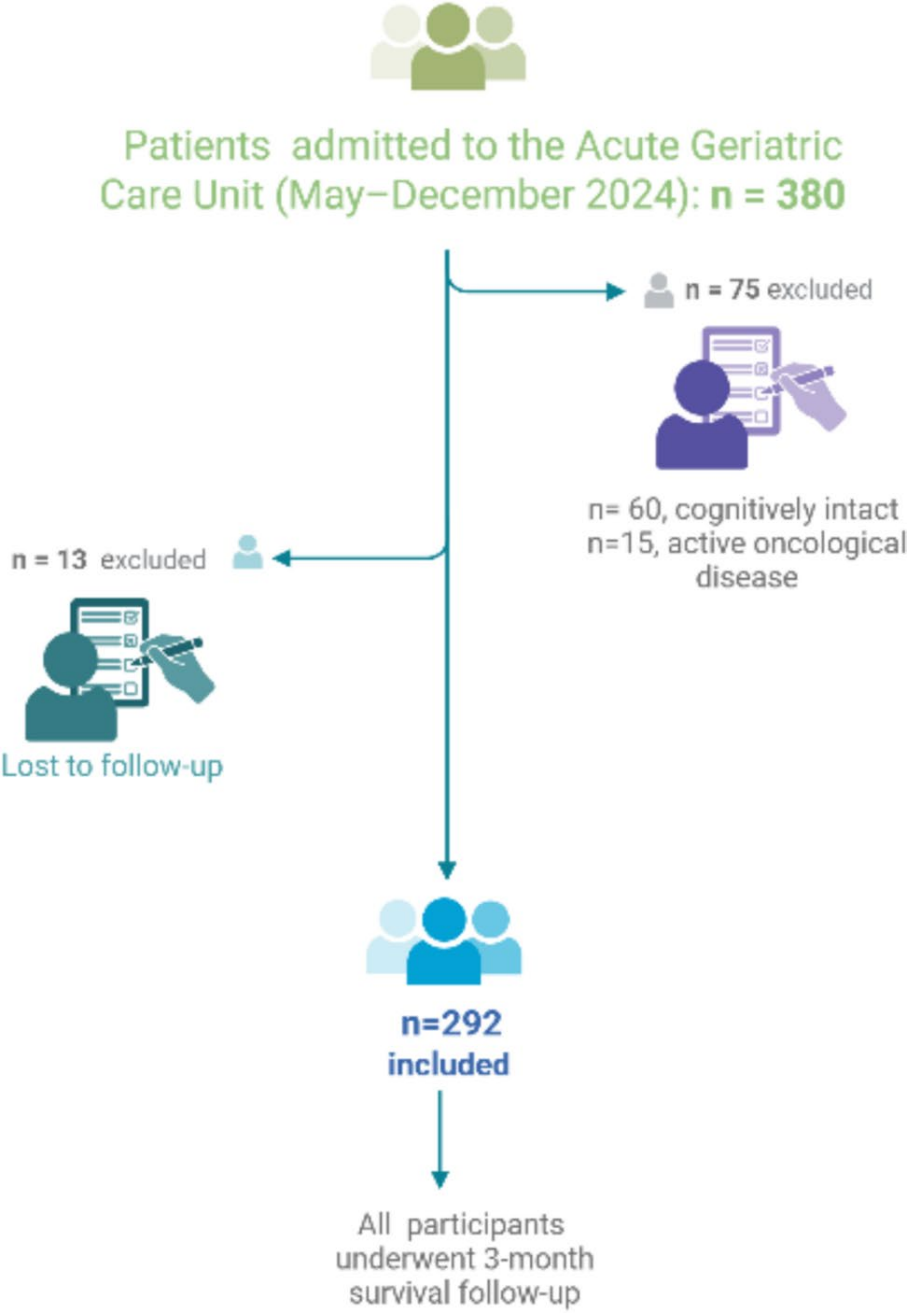
Flowchart of participant screening, exclusions, and inclusion in the study cohort

Participants were excluded if they were cognitively intact, had a diagnosis of active oncological disease, or had incomplete follow-up data. Of the 380 individuals screened, 292 fulfilled the inclusion criteria and were included in the final analysis (Fig. 1). The study was conducted in accordance with the Declaration of Helsinki and approved by the Ethics Committee of the University of Perugia (Prot. N. CE-685/24). Because many participants had moderate-to-severe cognitive impairment, written informed consent was obtained from legally authorized representatives, in accordance with ethical standards.

### Clinical and multidimensional assessment

Clinical information was obtained through structured history taking and comprehensive multidimensional geriatric assessment. All assessments were performed within the first 48 h of hospital admission by trained geriatric clinicians, according to standardized protocols. Data collection included demographic characteristics, cognitive and functional status, and pharmacological therapy. The clinical examination was complemented by the administration of scales aimed at defining short- and long-term adverse outcomes through the Activities of Daily Living (ADL), Instrumental Activities of Daily Living (IADL) scales [19, 20], cognitive status through the Short Portable Status Mental Questionnaire (SPSMQ) [21], nutritional status through the Mini Nutritional Assessment (MNA) scale [22], the presence of comorbidities through the Cumulative Illness Rating Scale (CIRS-G) [23], and polypharmacy therapy. Caregiver risk was also assessed through the Blaylock Risk Assessment Screening Score (BRASS) [24], frailty status through the Clinical Frailty Scale (CFS) [25] and quality of life through the EQ5D-3L Scale [26]. For the EQ-5D-3L, we used the descriptive system summary score, with higher scores indicating poorer health-related quality of life. The exclusive use of proxy respondents was chosen to ensure consistency of assessment across all dementia severity stages and to avoid potential bias related to unreliable self-reporting in the acute hospital setting. The quick Sequential Organ Failure Assessment (qSOFA) [27] was used to identify the severity of the patient’s clinical condition at the time of hospital admission. The qSOFA score is composed of three clinical parameters: systolic blood pressure ≤ 100 mmHg, respiratory rate ≥ 22 breaths/min, and altered mental status. Although originally developed for sepsis, qSOFA has been shown to predict short-term mortality in heterogeneous hospitalized populations, including older adults with acute medical conditions [28, 29].

### Pain assessment

Pain was evaluated by the PAIN-AD scale, an observational tool designed to assess pain in non-verbal older adults with advanced dementia. It evaluates five behavioral indicators: breathing (independent of vocalization), negative vocalizations (such as moaning or crying), facial expressions (like grimacing or frowning), body language (including rigidity or guarding), and consolability (the ability to be comforted). Each indicator is scored from 0 to 2, where 0 indicates no pain and 2 indicates high pain severity, yielding a total score ranging from 0 to 10. Higher scores reflect greater pain intensity. PAIN-AD assessment was performed during routine clinical evaluation within the first 48 h of hospital admission, by trained geriatric clinicians as part of standard care, with formal experience in dementia care. All assessors received specific training on the use of observational pain assessment tools prior to study initiation.

In line with previous validation studies, a PAIN-AD score ≥ 2 was considered indicative of probable pain, while a score of 1 was interpreted as a warning sign of discomfort potentially evolving into pain [12]. A cut-off value of 2 points on the total score is the most appropriate for identifying the presence of pain. A score of 1 is considered a warning sign of patient discomfort that could evolve into a pain situation. PAIN-AD was used to capture observable pain-related behaviours at the time of hospitalization and was not intended to establish a formal diagnosis of chronic pain, but rather to identify clinically relevant pain potentially requiring intervention in cognitively impaired patients. Although individuals with mild or moderate dementia may be able to self-report pain under stable conditions, acute hospitalization is frequently associated with communication difficulties, and fluctuating cognitive status. For this reason, PAIN-AD was selected to ensure a standardized assessment approach across all dementia severity stages.

### Analgesic therapy

Pharmacological pain management was defined as the prescription of systemic analgesic medications, including paracetamol, non-steroidal anti-inflammatory drugs, or opioids. Analgesic therapy was recorded separately at hospital admission and at discharge and was included as a covariate in the multivariable analyses. Non-pharmacological pain management strategies were not systematically documented and were therefore not considered in the present study.

### Statistical analysis

Continuous variables were tested for normal distribution using the Shapiro–Wilk test and are presented as means ± standard deviation (SD). Categorical variables are reported as absolute numbers and percentages. Comparisons between groups defined by PAIN-AD categories (≤ 1 vs ≥ 2) were performed using the independent-samples t test and the chi-square test for categorical variables. Pearson’s correlation analyses were used to explore bivariate associations between PAIN-AD scores and continuous clinical variables. Partial correlation analyses adjusted for age and sex were also conducted to account for potential confounding effects. Multivariable linear regression models were performed to investigate factors independently associated with frailty, assessed by the CFS, and with health-related quality of life, assessed by the EQ-5D-3L. Covariates included age, sex, nutritional status (by MNA), acute clinical severity (qSOFA score), analgesic therapy and dementia severity (CDR). Regression coefficients are reported as unstandardized B values with 95% confidence intervals (CIs). Length of hospital stay (LOS) was calculated as the difference in days between hospital discharge and admission dates. Patients who died during hospitalization, were transferred to another facility, or had missing discharge dates were excluded from LOS analyses. Given the longitudinal nature of the study and the availability of time-to-event data, the association between PAIN-AD and 3-month mortality was evaluated using Cox proportional hazards regression models. Hazard ratios (HRs) with 95% CIs were estimated after adjustment for multiple covariates. Survival curves were generated using the Kaplan–Meier method and compared using the log-rank (Mantel–Cox) test. Time zero was defined as the date of hospital admission, corresponding to study enrolment. All statistical tests were two-tailed, and a p value ≤ 0.05 was considered statistically significant. Statistical analyses were performed using SPSS software, version 26.0 (IBM Corp., Chicago, IL, USA).

## Results

A total of 292 participants (185 females and 107 males) admitted to the acute geriatric care unit were included in the study, with a mean age of 87.8 ± 6.2 years. The main causes of hospital admission included pneumonia and respiratory failure (n = 76, 26.0%), sepsis (n = 31, 10.6%), delirium (n = 30, 10.3%), falls and postural instability (n = 29, 9.9%), heart failure (n = 26, 8.9%), stroke (n = 16, 5.5%), and bowel obstruction (n = 14, 4.8%). The remaining 23.2% of admissions were due to other medical conditions. Of the 292 hospitalized patients, 110 (37.6%) had a PAIN-AD score ≤ 1, indicating no or minimal observable pain-related behaviours. In contrast, 182 patients (62.4%) exhibited a PAIN-AD score ≥ 2, consistent with probable pain. Despite this high prevalence, only 20.3% (n = 37) of patients with PAIN-AD ≥ 2 were receiving pharmacological analgesic treatment at the time of hospital admission. The distribution of PAIN-AD categories differed significantly across levels of dementia severity (χ^2^ = 20.99, p < 0.001). Clinically relevant pain (PAIN-AD ≥ 2) was progressively more frequent with increasing dementia severity, being observed in 89 of 170 patients (52.4%) with mild dementia (CDR 1), 59 of 83 (71.1%) with moderate dementia (CDR 2), and in the large majority of patients with more advanced dementia stages (CDR ≥ 3).

Baseline characteristics stratified by PAIN-AD categories (≤ 1 vs ≥ 2) are reported in Table 1. The overall mean length of hospital stay in the study population was 8.8 ± 5.5 days. Age and sex distribution were comparable between groups. Participants with PAIN-AD ≥ 2 showed significantly lower functional autonomy, as reflected by reduced ADL and IADL scores, and poorer nutritional status as assessed by the MNA. Clinically relevant pain was also associated with higher frailty levels, measured by the CFS, and greater acute clinical severity, as indicated by higher qSOFA scores. In addition, participants with PAIN-AD ≥ 2 exhibited significantly higher BRASS scores, suggesting a greater complexity of discharge planning and post-acute care needs. Participants with PAIN-AD ≥ 2 also showed worse cognitive performance (SPMSQ), whereas no significant differences were observed in comorbidity burden (CIRS-G) or number of prescribed medications. Health-related quality of life, assessed by the EQ-5D-3L, tended to be worse in participants with PAIN-AD ≥ 2, although this difference did not reach statistical significance. Finally, participants with clinically relevant pain experienced a significantly longer length of hospital stay compared with those with PAIN-AD ≤ 1. Accordingly a positive correlation was observed between PAIN-AD scores and length of hospital stay (r = 0.205, p = 0.001).

**Table 1:** Descriptive statistics in all population and according to PAIN-AD categories

	Total (n = 292)	PAIN-AD ≤ 1 (n = 110)	PAIN-AD ≥ 2 (n = 182)	p-value
Age, years	87.8 ± 6.2	87.6 ± 5.7	87.9 ± 6.5	0.111
Sex, F/M	185/107	64/46	121/61	0.097
ADL score	2.2 ± 2.0	3.2 ± 2.1	1.6 ± 1.7	< 0.001
IADL score	1.5 ± 2.1	2.3 ± 2.2	0.9 ± 1.7	< 0.001
SPMSQ (Short) score	4.0 ± 3.4	5.7 ± 3.2	3.0 ± 3.1	< 0.001
MNA score	17.3 ± 8.28	19.59 ± 6.47	15.85 ± 8.93	< 0.001
BRASS score	17.4 ± 6.5	14.2 ± 5.5	19.6 ± 6.2	< 0.001
CIRS-G score	9.7 ± 5.3	9.5 ± 5.3	9.9 ± 5.4	0.948
qSOFA score	1.5 ± 0.6	1.3 ± 0.5	1.6 ± 0.7	< 0.001
CFS score	6.8 ± 2.0	6.4 ± 2.2	7.4 ± 1.7	0.019
EQ-5D-3L score	9.4 ± 3.3	8.4 ± 3.5	10.3 ± 2.9	0.064
Number of drugs, n	7.0 ± 3.0	7.0 ± 3.2	7.0 ± 2.9	0.240
LOS days	8.8 ± 5.5	7.2 ± 4.7	9.7 ± 5.8	< 0.001

Continuous variables are reported as mean ± standard deviation and were compared between groups using unpaired t test. Categorical variables are presented as counts and percentages and were compared using the chi-square test

*ADL* Activities of Daily Living, *IADL* Instrumental Activities of Daily Living, *SPMSQ* Short Portable Mental Status Questionnaire, *MNA* Mini Nutritional Assessment, *BRASS* Blaylock Risk Assessment Screening Score, *CIRS-G* Cumulative Illness Rating Scale-Geriatric, *qSOFA* quick Sequential Organ Failure Assessment, *CFS* Clinical Frailty Scale, *EQ-5D-3L* EuroQol 5-Dimension 3-Level questionnaire, *LOS* length of stay

### PAIN-AD, frailty, and quality of life

Significant bivariate correlations were observed between PAIN-AD scores and the CFS (r = 0.297, p ≤ 0.001), as well as between PAIN-AD scores and EQ-5D-3L (r = 0.318, p ≤ 0.001). These correlations remained statistically significant after adjustment for age and sex, both for frailty (partial r = 0.266, p = 0.001) and quality of life (partial r = 0.292, p ≤ 0.001). To further explore these associations, a multivariable linear regression model was performed with frailty, assessed by the CFS, as the dependent variable, adjusting for age, sex, dementia severity (CDR), analgesic therapy at admission, qSOFA score, nutritional status (MNA), and pain severity (PAIN-AD) (Table 2). In this model, both PAIN-AD (B = 0.237, 95% CI 0.068–0.407, p = 0.006) and MNA (B = − 0.075, 95% CI − 0.121 to − 0.028, p = 0.002) remained independently associated with frailty. Female sex was also independently associated with higher CFS scores (B = 0.733, 95% CI 0.074–1.391, p = 0.029). In contrast, age, dementia severity (CDR), qSOFA score, and analgesic therapy at admission were not independently associated with frailty in the fully adjusted model.

**Table 2:** Multivariable linear regression analysis exploring factors associated with frailty (CFS)

	B	95% CI	p value
Age (years)	0.035	− 0.022 to 0.092	0.232
Sex (female vs male)	0.733	0.074–1.391	0.029
qSOFA score	0.299	− 0.301 to 0.898	0.326
Mini Nutritional Assessment (MNA)	− 0.075	− 0.121 to − 0.028	0.002
PAIN-AD score	0.237	0.068–0.407	0.006
Analgesic therapy at admission	0.731	− 0.034 to 1.496	0.061
Clinical Dementia Rating (CDR)	0.233	− 0.184 to 0.651	0.271

Model R^2^ = 0.268; Adjusted R^2^ = 0.226

Dependent variable: Clinical Frailty Scale (CFS)

Sex coded as male = 0, female = 1; analgesic therapy coded as no = 0, yes = 1

A similar multivariable linear regression model was conducted using health-related quality of life, assessed by the EQ-5D-3L, as the dependent variable, adjusting for age, sex, dementia severity (CDR), analgesic therapy at admission, qSOFA score, nutritional status (MNA), and pain severity (PAIN-AD) (Table 3). In this fully adjusted model, PAIN-AD (B = 0.449, 95% CI 0.169–0.730, p = 0.002) and MNA (B = − 0.104, 95% CI − 0.182 to − 0.027, p = 0.008) remained independently associated with EQ-5D-3L scores. CDR was also independently associated with worse health-related quality of life (B = 0.845, 95% CI 0.152–1.537, p = 0.017). In contrast, age, sex, qSOFA score, and analgesic therapy at admission did not reach statistical significance in the adjusted analysis. The same analysis with length of hospital stay as the dependent variable, showed that higher PAIN-AD scores are independently associated with longer LOS (B = 0.49, 95% CI 0.14–0.84; p = 0.007; data not shown).

**Table 3:** Multivariable linear regression analysis exploring factors associated with health-related quality of life (EQ-5D-3L)

	B	95% CI	p value
Age (years)	0.089	− 0.006 to 0.184	0.065
Sex (female vs male)	0.965	− 0.127 to 2.057	0.083
qSOFA score	0.601	− 0.393 to 1.595	0.233
Mini Nutritional Assessment (MNA)	− 0.104	− 0.182 to − 0.027	0.008
PAIN-AD score	0.449	0.169–0.730	0.002
Analgesic therapy at admission	0.911	− 0.357 to 2.180	0.158
Clinical Dementia Rating (CDR)	0.845	0.152–1.537	0.017

Model R^2^ = 0.300; Adjusted R^2^ = 0.259

Dependent variable: EQ-5D-3L score

Sex coded as male = 0, female = 1; analgesic therapy coded as no = 0, yes = 1

### PAIN-AD and short-term mortality

At 3 months following hospital discharge, a total of 91 patients (31.2%) had died. Mortality differed markedly according to pain severity, occurring in 18 of 110 patients (16.3%) with PAIN-AD scores ≤ 1 and in 73 of 182 patients (40.1%) with PAIN-AD scores ≥ 2. Among survivors, 34.3% experienced at least one rehospitalization during the same follow-up period.

In the multivariable Cox proportional hazards regression analysis assessing 3-month mortality, clinically relevant pain (PAIN-AD ≥ 2) remained independently associated with a significantly increased risk of death (HR 2.39, 95% CI 1.20–4.74, p = 0.013), after adjustment for age, sex, dementia severity, nutritional status, qSOFA score, and analgesic therapy at discharge. Nutritional status showed a protective trend but did not reach statistical significance in the fully adjusted model (HR 0.97, 95% CI 0.94–1.00, p = 0.092). Age and female sex exhibited borderline associations with mortality, whereas qSOFA score, dementia severity, and analgesic therapy at discharge were not significantly associated with mortality in the adjusted analysis (Table 4).

**Table 4:** Multivariable Cox proportional hazards regression analysis for 3-month mortality (n = 292)

	HR	95% CI	p value
Age (years)	1.04	1.00–1.09	0.056
Sex (F vs M)	0.60	0.36–1.01	0.054
qSOFA score	1.33	0.89–1.99	0.158
MNA score	0.97	0.94–1.00	0.092
PAIN-AD ≥ 2 (vs ≤ 1)	2.39	1.20–4.74	0.013
Analgesic therapy at discharge (yes vs no)	1.44	0.86–2.41	0.166
CDR score	1.24	0.90–1.70	0.190

χ^2^ = 33.56, p < 0.001

*HR* hazard ratio, *CI* confidence interval, *PAIN-AD* Pain Assessment in Advanced Dementia, *MNA* Mini Nutritional Assessment, *CDR* Clinical Dementia Rating

**Fig. 2:**
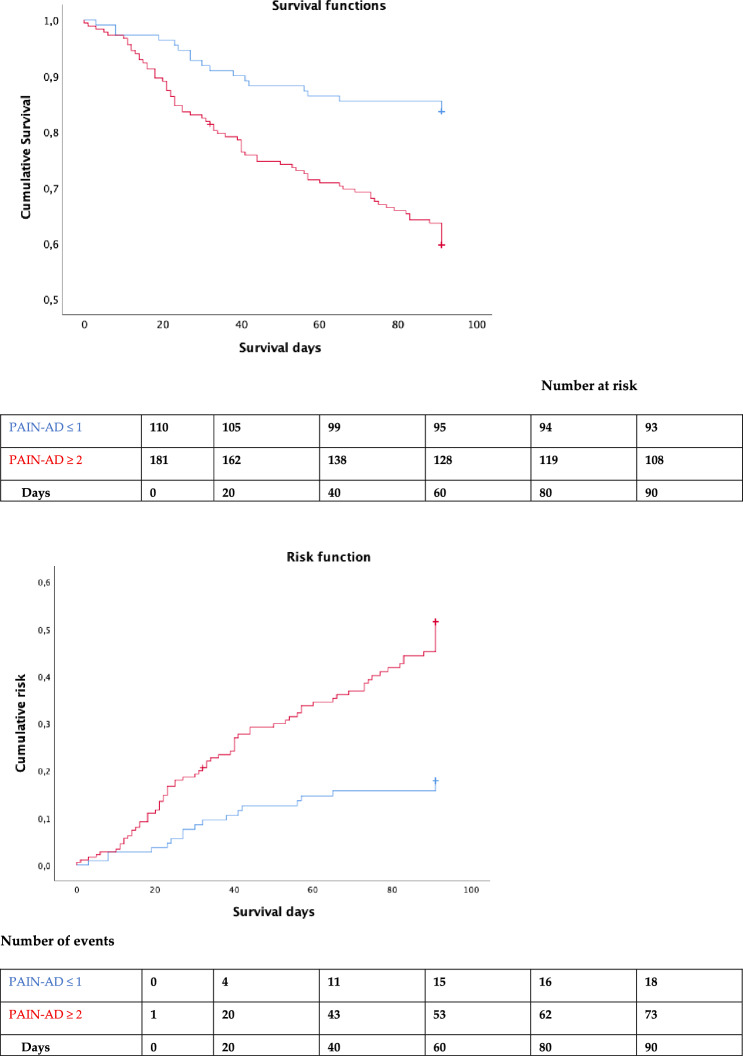
Cumulative survival and risk functions in patients with dementia, stratified by PAIN-AD scores

To visually illustrate these findings, Kaplan–Meier survival analysis was performed according to PAIN-AD categories. As shown in Fig. 2, patients with PAIN-AD scores ≤ 1, indicating minimal or absent pain-related behaviours, exhibited significantly longer survival compared with those with PAIN-AD scores ≥ 2. The difference between survival curves was statistically significant, as confirmed by the log-rank test (χ^2^ = 17.188, p < 0.001).

One participant in the PAIN-AD ≥ 2 group experienced the event at time zero; therefore, the number at risk at Day 0 is 181.

## Discussion

Collectively, our findings highlight the underrecognized impact of pain in older adults with dementia and its association with adverse clinical outcomes, including poorer quality of life and increased mortality. The study demonstrates that higher PAIN-AD scores are significantly associated with frailty (CFS), lower health-related quality of life (EQ-5D-3L), and an increased risk of short-term mortality, even after adjusting for relevant confounders. Although the magnitude of these associations was modest, their consistency across multiple clinically meaningful domains—functional vulnerability, discharge complexity, quality of life, hospitalization burden, and survival—supports the clinical relevance of pain-related behaviours in acute geriatric care. In this study, pain is conceptualized as a clinically relevant geriatric syndrome, while the PAIN-AD scale is used as a standardized observational instrument to identify pain-related behaviours in patients unable to self-report. Importantly, the aim was not to validate the PAIN-AD scale per se, but to investigate whether observable pain-related behaviours carry prognostic significance in a real-world acute geriatric setting.

In our cohort, despite the high prevalence of pain (62.4% with PAIN-AD ≥ 2), pharmacological pain management was markedly suboptimal, with only 20.3% of these patients receiving analgesic treatment at hospital admission. This mirrors findings from previous studies indicating that pain in dementia is often undertreated due to difficulties in self-reporting and clinical underrecognition [30], particularly in acute hospital settings where behavioural symptoms may be attributed to acute illness rather than pain [31, 32]. This gap between observed pain-related behaviours and analgesic prescription underscores a potentially modifiable aspect of acute geriatric care. The observed association between PAIN-AD scores and frailty aligns with prior studies linking chronic pain to increased frailty risk in older adults. Evidence suggests that persistent pain contributes to both the onset and progression of frailty, independent of sociodemographic and health-related confounders [33]. Our findings extend this evidence to hospitalized patients with major neurocognitive disorders, showing that higher levels of observable pain-related behaviours are associated with greater frailty severity even after accounting for age, sex, dementia severity, acute illness severity, and nutritional status. This supports the hypothesis that inadequately recognized pain may act as a stressor amplifying vulnerability in acutely ill older adults. Moreover, the strong association between PAIN-AD and EQ-5D-3L suggests that pain significantly impacts the perceived quality of life in this population. These findings are consistent with previous research demonstrating that untreated pain is a major contributor to reduced well-being in older adults with neurocognitive disorders. For instance, Achterberg et al. [34] found that pain was a leading determinant of reduced quality of life in nursing home residents with dementia, as measured by EQ-5D-3L and other multidimensional assessment tools. Our results extend these observations to the acute hospital setting, indicating that pain-related behaviours remain associated with poorer health-related quality of life even in the presence of severe comorbidity and acute illness.

Beyond functional and quality-of-life outcomes, our study provides evidence of an association between pain-related behaviours and short-term mortality. In the Cox proportional hazards model, PAIN-AD ≥ 2 remained independently associated with increased 3-month mortality after adjustment for demographic variables, dementia severity, nutritional status, acute clinical severity, and analgesic therapy. Previous literature has reported mixed findings regarding the relationship between pain and mortality in older adults. A previous review [35] suggested that higher pain intensity in dementia patients may be associated with increased mortality over longer follow-up periods. Moreover, evidence from large population-based cohorts indicates that pain interference with daily life, rather than pain extent alone, is associated with increased mortality risk [8]. In this context, PAIN-AD may capture a phenotype of pain-related distress and functional impact that is closer to “pain interference” than to pain presence alone, potentially explaining its prognostic relevance. The Kaplan–Meier survival analysis further supports a temporal association between pain-related behaviours and reduced survival, with patients exhibiting PAIN-AD ≥ 2 showing significantly lower short-term survival. While causality cannot be inferred, this temporal pattern reinforces the hypothesis that pain-related behaviours may serve as an early clinical marker of vulnerability and adverse prognosis in hospitalized older adults with dementia. However, these findings should be interpreted with caution, as several established prognostic factors in dementia (such as behavioural and psychological symptoms, swallowing disorders, and other markers of acute and chronic disease burden) were not systematically collected and could not be included, leaving residual confounding.

To our knowledge, this is among the first prospective studies to demonstrate that observationally assessed pain-related behaviours are independently associated with short-term mortality in hospitalized older adults with major neurocognitive disorder. This study has several strengths, along with limitations that should be acknowledged. A key strength is its comprehensive multidimensional assessment evaluating pain-related behaviours, frailty, quality of life, and multimorbidity in older adults with dementia. The relatively large sample size provides adequate statistical power, while multivariable regression models controlling for potential confounders enhance the robustness of the findings. Additionally, the inclusion of survival analysis strengthens the evidence linking pain-related behaviours to short-term mortality. Although the longitudinal design allows the assessment of temporal associations, causal relationships cannot be inferred due to the observational nature of the study. Pain was assessed at a single time point, despite its dynamic nature, representing an important limitation. Potential measurement bias may arise from reliance on behavioural indicators rather than self-report. Important clinical predictors of mortality in dementia—such as behavioural and psychological symptoms, swallowing disorders, and pressure ulcers—were not systematically collected and could not be included in the multivariable models, raising the possibility of residual confounding. Indeed, assessment instruments used have not undergone formal validation within the target linguistic and cultural context. This may introduce measurement bias and compromise the reliability and interpretability of the findings.

## Conclusions and implications

This study highlights pain as an underrecognized and clinically meaningful geriatric syndrome in hospitalized older adults with dementia. Observable pain-related behaviours, as assessed by PAIN-AD, were consistently associated with increased frailty, greater discharge complexity, longer hospital stay, reduced quality of life, and elevated short-term mortality risk. Although these associations were modest in magnitude, their consistency across multiple clinically relevant outcomes supports the importance of systematic pain assessment in acute geriatric care. Structured implementation of observational pain assessment tools, combined with individualized pain management strategies, may represent an important opportunity to improve care quality and short-term outcomes in this highly vulnerable population.

## Data Availability

The data supporting the findings of this study are available from the corresponding author.
